# Very Early Levodopa May Prevent Self-Injury in Lesch-Nyhan Disease

**DOI:** 10.1016/j.pediatrneurol.2024.03.020

**Published:** 2024-03-26

**Authors:** Jasper E. Visser, Odelia Chorin, H.A. Jinnah

**Affiliations:** a Department of Neurology, Donders Institute for Brain, Cognition and Behavior, Radboud University Medical Center, Nijmegen, The Netherlands; b Faculty of Science, Donders Institute for Brain, Cognition and Behavior, Radboud University Medical Center, Nijmegen, The Netherlands; c Department of Neurology, Amphia Hospital, Breda, The Netherlands; d The Institute of Rare Diseases, Lily and Edmond Safra Children’s Hospital, Sheba Medical Center, Tel Hashomer, Israel; e Departments of Neurology, Human Genetics, & Pediatrics, Emory University School of Medicine, Atlanta, Georgia

**Keywords:** Lesch-Nyhan disease, Levodopa, Dopamine, Self-injury, Automutilation, Dystonia, Treatment, Brain development

## Abstract

**Background:**

In Lesch-Nyhan disease (LND), early dopamine deficiency is thought to contribute to dystonia and self-injury, gradually developing over the first years of life. Previous attempts to restore dopamine levels in older patients have been unsuccessful. Based on the hypothesis that *very early* dopamine replacement can *prevent* full phenotypic development, we treated three patients with LND from infancy with levodopa.

**Methods:**

Levodopa/carbidopa (4:1) was started at age 11 to 13 months, aiming at escalating to 5 to 6 mg/kg levodopa per day. Follow-up focused on dystonia severity and whether self-injury occurred. In addition, the literature was reviewed to delineate the age at onset of self-injury for all reported cases to date.

**Results:**

During long-term follow-up, self-injury appears to have been prevented in two patients (now aged 14 and 15.5 years), as their *HPRT1* gene mutations had been invariably associated with self-injury before. Future self-injury is unlikely, as only 1.1% of 264 published cases had self-injury onset later in life than these patients’ current ages. The third patient started self-injury at age 1.5 years, while on a substantially lower levodopa dose. A clear effect of levodopa on dystonia could not be determined.

**Conclusions:**

Our observations suggest that levodopa, given early enough and sufficiently dosed, might be able to prevent self-injury in LND. Therefore, levodopa could be considered in patients with LND as early as possible, at least before the self-injury appears. Further research is needed to establish very early levodopa as an effective treatment strategy in LND, and to optimize timing and dosing.

## Background

Lesch-Nyhan disease (LND) is caused by pathogenic variants in the *HPRT1* gene encoding the purine salvage enzyme hypoxanthine-guanine phosphoribosyl transferase (HGprt).^1,2^ Absence of HGprt causes hyperuricemia and a neurobehavioral phenotype characterized by dystonia, cognitive deficits, and behavioral abnormalities including self-injury.^2,3^ Usually, the movement disorder prevents ambulation, and patients require assistance with all activities of daily living.^3^ Irresistible self-injury is the most troublesome aspect of LND. Patients often bite lips and fingers, poke eyes, and hit limbs against sharp objects, resulting in severe tissue damage if not prevented.^4^

HGprt deficiency is associated with a spectrum of clinical severity. Near-complete absence of HGprt activity results in the classic LND phenotype with self-injury. *HPRT1* pathogenic variants allowing residual HGprt enzyme activity may cause partial phenotypes without self-injury. Patients with LND are usually born apparently healthy, developing their clinical phenotype within the first years of life. Self-injurious behavior starts typically before age four years, although it may start later. Effective therapies for LND are currently lacking. For some symptomatic relief, patients may use muscle relaxants and benzodiazepines.^3,5^ Self-injury is usually only prevented by physical restraints and extraction of teeth.^2,5^

The exact mechanisms by which HGprt deficiency leads to neurobehavioral abnormalities are unknown. Clinical and experimental data provide strong support that abnormal development of brain dopamine pathways provides the neuropathologic basis of LND, associated with a dopamine deficit.^6–9^ Nevertheless, increasing dopamine with its precursor levodopa has not been successful in patients with established phenotypes. In a prospective open-label dose-escalation study, all participants discontinued levodopa early, often due to worsening of motor function.^10^ Self-injury appeared unchanged, possibly explained by short treatment duration or lower sensitivity to levodopa. Based on phenotypical similarities with levodopa-induced dyskinesias and impulse control disorders in Parkinson disease—due to presynaptic and postsynaptic adaptive changes in dopaminergic circuitry after profound nigral degeneration—it was speculated that similar neural adaptive processes after early dopamine deficiency is an essential mechanism in LND pathogenesis.^10^

The concept that the age at which dopamine depletion occurs profoundly influences motor and behavioral outcome, as well as response to levodopa, is supported by experimental studies. For example, destruction of nigrostriatal dopamine neurons in adult rats produces a motor syndrome resembling parkinsonism, ameliorated by levodopa. However, the same lesion in neonatal rats causes hyperactivity, exacerbated by levodopa.^11^ Moreover, only in neonatally lesioned animals dopaminergic drugs can elicit aggression and self-injurious biting, suggesting that dopaminergic circuitry may also be involved in these difficult behaviors observed in LND.^11^

Based on the hypothesis that early dopamine deficiency in LND induces neural adaptation that is crucial for developing the clinical phenotype, we attempted to restore dopamine levels in three infants with LND to *prevent* these neuroplastic changes. After long-term follow-up, this approach appears to have significantly improved the clinical outcome in two patients. Factors that may have influenced outcome may further guide the application of very early levodopa as a treatment option for LND.

## Patients

Patient A had poor head control at age four months, progressing to generalized hypotonia and dyskinesias at 7.5 months. LND was suspected based on elevated blood and urine uric acid, confirmed by a *HPRT1* c.151C>T; p.R51X pathogenic nonsense mutation. At our examination at 12 months, dystonia was noticed in the face, trunk, and extremities. At rest, hypotonia was noted. There were no signs of self-injury. Levodopa/carbidopa (ratio 4:1) was then started, escalating up to ~6 mg/kg/day levodopa divided in three doses (Fig A). During follow-up, up to age 15.5 years, the patient never started self-injury. Persistent dystonia (Fig B) and hypotonia prevent sitting independently, and hand functionality is limited. Clonazepam reduces dystonic movements, ballismus, and stress.

Patient B started physiotherapy at age four months, because of stiffness and difficulty keeping his head upright. Kidney stones and hyperuricemia suggested the diagnosis of LND, confirmed by absent erythrocyte HGprt activity and a *HPRT1* c.222C>G; p.F74L pathogenic variant. Upon examination at 12 months, generalized dystonia was present and hypotonia was present at rest. The boy could roll over, but not sit unassisted. At age 13 months, levodopa/carbidopa (ratio 4:1) was started, escalating up to ~6 mg/kg/day levodopa divided in three doses (Fig A). Until now, at age 14 years, no signs of self-injurious behavior appeared, albeit generalized dystonia remained (Fig B). Nevertheless, he mobilizes by crawling, stands and walks supported, and sits independently. There is some functional hand use.

Patient C showed restlessness at age two weeks and irritability with episodes of hyperextension at 2 months. At six months, orange diaper stains suggested urine uric acid crystals due to hyperuricemia. LND was confirmed by a previously unreported *HPRT1* c.202C>T; p.L68F variant. Levodopa/carbidopa (ratio 4:1) was started at 11 months, gradually increasing to ~5 mg/kg/day levodopa in three doses (Fig A). Dystonia, chorea, and restlessness subsequently worsened during a *roseola infantum* infection. After reducing levodopa by half, these exacerbated motor symptoms subsided. While continuing on ~2.5 mg/kg/day levodopa, self-injury started at age 1.5 years with biting fingers and inner cheeks. To prevent self-injury, the patient wears gloves.

## Discussion

Currently, there are no effective treatments available for any of the neurobehavioral disturbances in LND. Previous attempts to restore dopamine levels in older patients were unsuccessful.^10^ We hypothesized that very early levodopa, i.e., starting before symptoms have fully developed, may be beneficial by preventing assumed adaptive neural changes caused by the dopamine deficit. The gradual development of clinical features early in life suggests a therapeutic window of opportunity for such preventive treatment—a concept that might explain the anecdotal improvement in a single patient with LND treated with levodopa from age 10 months.^12^ We attempted to restore dopamine levels in three infants with LND, starting at age 11 to 13 months, aiming at escalating up to 5 to 6 mg/kg per day in divided doses, as suggested for other inborn dopamine deficiencies.^13^

We noted that levodopa was generally better tolerated at these young ages than previously in older patients.^10^ Patient C developed motor side effects, but a simultaneous viral infection may have contributed to this deterioration as well—particularly because levodopa was better tolerated at a second escalation attempt.

Although these individual observations do not allow definitive conclusions regarding efficacy of very early levodopa in LND, we believe that this approach has prevented the development of self-injury in Patients A and B, for several reasons. First, the *HPRT1* pathogenic variants in these patients have been invariably associated with self-injury in prior literature (available at www.leschnyhan.org). The genetic variant of Patient A, causing early truncation of *HPRT1* RNA, complete absence of HGprt activity, and a full clinical phenotype with reported onset of self-injury of age four years, has been described over 20 times.^14^ The genetic variant of Patient B has been previously described to cause self-injury in two patients, at age two and six years.^15^ In addition, genetic variants in six other patients causing the same amino acid substitution (p.F74L) have been invariably linked to self-injury starting considerably earlier than current age of Patient B. Taken together, these prior reports render future self-injury in patients A and B very unlikely. To further substantiate this interpretation, we reviewed the literature to delineate age at onset of self-injury in LND (Fig C). Self-injury has been reported for 264 cases to date, starting between age six months and 20 years, with a median of 2.0 years. At age eight years, 95% of patients have started self-injury. Only three cases (1.1%) had self-injury onset later in life than the current ages of Patients A and B.

Levodopa did not prevent self-injury in Patient C. A plausible explanation might be that the levodopa dose was insufficient, being less than 50% compared with the other patients (Fig A). Alternatively, the time window of opportunity for Patient C might have been earlier than for the others, as some patients start self-injury extremely young (Fig C).

The movement disorder in Patients A and B seemed less affected by levodopa than the self-injury, possibly explained by a different optimal therapeutic time window or a different sensitivity to levodopa for preventing adaptive changes in motor compared with behavioral neuronal circuitry. Of note, Patient B gained better motor functionality than Patient A, perhaps due to an initially higher levodopa dose (Fig A) or due to phenotypic variability, as Patient B had a lower dystonia severity at baseline (Fig B).^3^

In summary, our observations suggest that levodopa may prevent self-injury in LND, if given early enough and dosed sufficiently. It remains unclear whether levodopa can also improve motor function. Therefore, we believe that levodopa could be considered in LND patients as early in life as possible, at least before the self-injury appears. Consequently, all infants with developmental delay and high uric acid levels should have prompt *HPRT1* gene mutation analysis to minimize the time to diagnosis. Rationally, levodopa/carbidopa (ration 4:1) could be escalated from 1 mg/kg/day levodopa in divided doses to at least 5 to 6 mg/kg/day, while carefully monitoring side effects. Side effects might prompt to temporarily lower the levodopa dose and escalate again as soon as possible at a lower speed. Higher doses of levodopa, if tolerated, could be considered to maximize the preventive effects of levodopa.

## Figures and Tables

**FIGURE.**
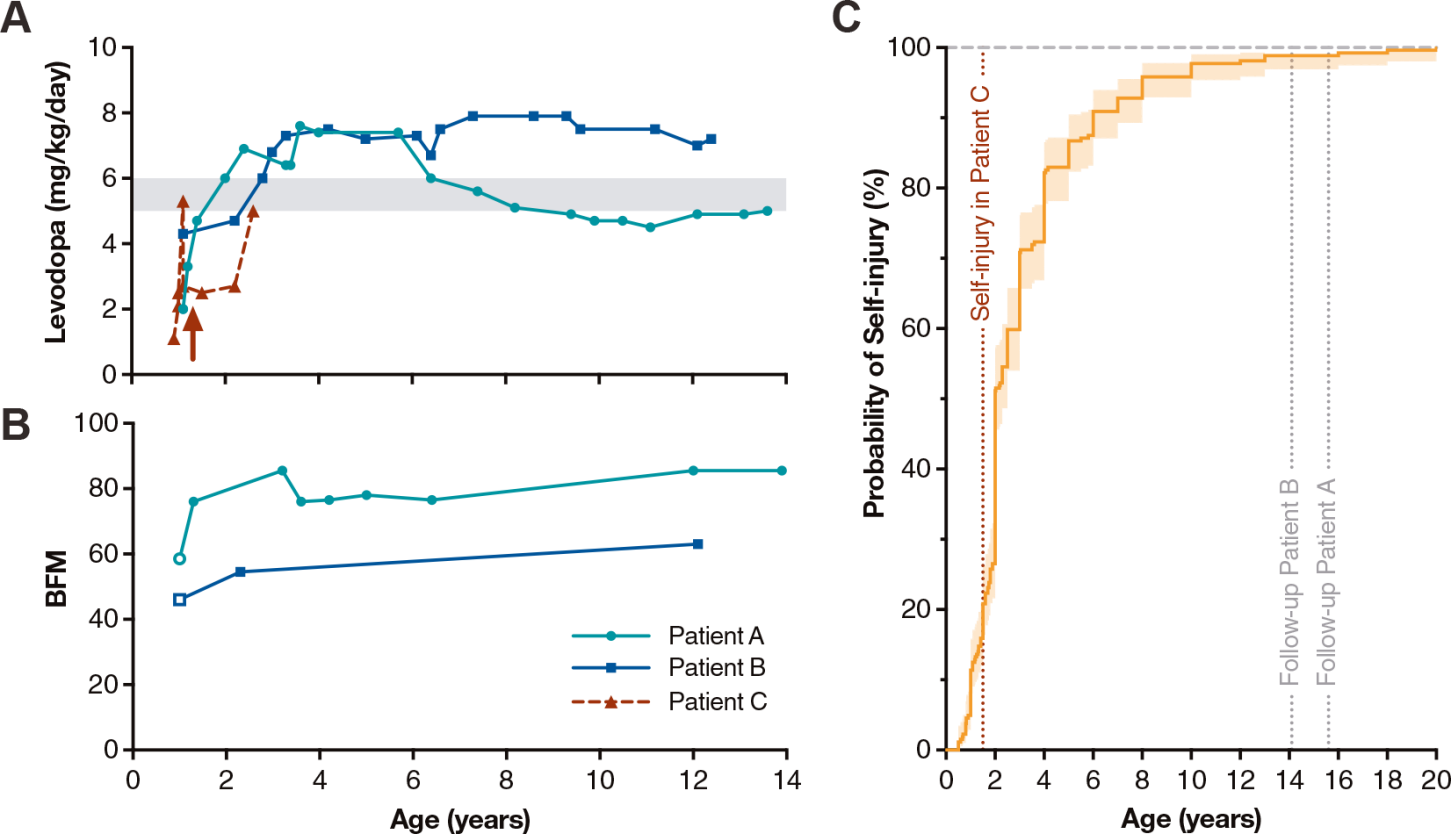
(A) Prescribed levodopa dose against age for Patients A to C with Lesch-Nyhan disease (LND). Shaded area indicates the initial target dose of 5 to 6 mg/kg/day levodopa. Red arrow indicates onset of self-injury in Patient C. (B) Severity of dystonia over time, as assessed by the Burke-Fahn-Marsden scale (BFM), for Patients A and B. Open symbols indicate assessments before levodopa started; closed symbols indicate assessments while using levodopa. For Patient C, insufficient BFM scores were collected to determine effects. (C) Kaplan-Meier survival curve indicating age of onset of self-injury in 264 patients with LND reported in the literature to date, starting between age six months and 20 years, with a median of 2.0 years. A chance of self-injury of 90% and 95% corresponds with ages six and eight years, respectively. Vertical dotted lines indicate ages at last follow-up for Patients A and B (gray), i.e., both beyond the age that 95% of patients developed self-injury, and the onset of self-injury in Patient C (red). Shaded area indicates 95% confidence interval.
